# Cardiometabolic Disorders and the Risk of Critical COVID-19 as Compared to Influenza Pneumonia

**DOI:** 10.3390/jcm10194618

**Published:** 2021-10-08

**Authors:** Antoine Fayol, Marine Livrozet, Héléna Pereira, Jean-Luc Diehl, David Lebeaux, Jean-Benoit Arlet, Bernard Cholley, Claire Carette, Jean-Baptiste Carves, Sébastien Czernichow, Caroline Hauw, Sophie-Rym Hamada, Anne-Sophie Jannot, Geoffroy Volle, Nihar Masurkar, Tristan Mirault, Benjamin Planquette, Olivier Sanchez, Gilles Châtellier, Michel Azizi, Jean-Sébastien Hulot

**Affiliations:** 1 Université de Paris, F-75006 Paris, France; antoine.fayol@aphp.fr (A.F.); marine.livrozet@aphp.fr (M.L.); helena.pereira@aphp.fr (H.P.); jean-luc.diehl@aphp.fr (J.-L.D.); david.lebeaux@aphp.fr (D.L.); jean-benoit.arlet@aphp.fr (J.-B.A.); bernard.cholley@aphp.fr (B.C.); claire.carette@aphp.fr (C.C.); jean-baptiste.carves@aphp.fr (J.-B.C.); sebastien.czernichow@aphp.fr (S.C.); caroline.hauw-berlemont@aphp.fr (C.H.); sophie.hamada@aphp.fr (S.-R.H.); annesophie.jannot@aphp.fr (A.-S.J.); geoffroy.volle@aphp.fr (G.V.); nihar.m1990@gmail.com (N.M.); tristan.mirault@aphp.fr (T.M.); benjamin.planquette@aphp.fr (B.P.); olivier.sanchez@aphp.fr (O.S.); gilles.chatellier@aphp.fr (G.C.); michel.azizi@aphp.fr (M.A.); 2 CIC1418 and DMU CARTE, Assistance Publique Hôpitaux de Paris (AP-HP), Hôpital Européen Georges-Pompidou, F-75015 Paris, France; 3 INSERM, Paris Cardiovascular Research Center, PARCC, UMR970, F-75015 Paris, France; 4 Clinical and Epidemiological Unit, AP-HP, Hôpital Européen Georges-Pompidou, F-75015 Paris, France; 5 Medical Intensive Care Unit, AP-HP, Hôpital Européen Georges-Pompidou, F-75015 Paris, France; 6 Service de Microbiologie, Unité Mobile d’Infectiologie, AP-HP, Hôpital Européen Georges-Pompidou, F-75015 Paris, France; 7 Internal Medicine Department, Sickle Cell Referral Center, AP-HP, Hôpital Européen Georges-Pompidou, F-75015 Paris, France; 8 Department of Anesthesia and Intensive Care, AP-HP, Hôpital Européen Georges-Pompidou, F-75015 Paris, France; 9 Service de Nutrition, Centre Spécialisé Obésité, AP-HP, Hôpital Européen Georges-Pompidou, F-75015 Paris, France; 10 Médecine Vasculaire HyperVASC and DMU CARTE, AP-HP, Hôpital Européen Georges-Pompidou, F-75015 Paris, France; 11 Service de Pneumologie et Soins Intensifs, AP-HP, INSERM UMR-S 1140, Hôpital Européen Georges-Pompidou, F-75015 Paris, France; 12 Hypertension Unit and DMU CARTE, AP-HP, Hôpital Européen Georges-Pompidou, F-75015 Paris, France

**Keywords:** COVID-19, seasonal influenza, cardiovascular risk factors, obesity, hypertension

## Abstract

We aimed to compare the influence of cardiometabolic disorders on the incidence of severe COVID-19 vs. non-COVID pneumonia. We included all consecutive patients admitted with SARS-CoV-2-positive pneumonia between 12 March 2020 and 1 April 2020 and compared them to patients with influenza pneumonia hospitalized between December 2017 and December 2019 at the same tertiary hospital in Paris. Patients with COVID-19 were significantly younger and more frequently male. In the analysis adjusted for age and sex, patients with COVID-19 were more likely to be obese (adjOR: 2.25; 95% CI 1.24–4.09; *p* = 0.0076) and receive diuretics (adjOR: 2.13; 95% CI 1.12–4.03; *p* = 0.021) but were less likely to be smokers (adjOR: 0.40; 95% CI 0.24–0.64; *p* = 0.0002), have COPD (adjOR: 0.25; 95% CI 0.11–0.56; *p* = 0.0008), or have a previous or active cancer diagnosis (adjOR: 0.54, 95% CI 0.32–0.91; *p* = 0.020). The rate of ICU admission was significantly higher in patients with COVID-19 (32.4% vs. 5.2% *p* < 0.0001). Obesity was significantly associated with the risk of direct ICU admission in patients with COVID-19 but not in patients with influenza pneumonia. Likewise, pre-existing hypertension was significantly associated with mortality in patients with COVID-19 but not in patients with influenza pneumonia. Cardiometabolic disorders differentially influenced the risk of presenting with severe COVID-19 or influenza pneumonia.

## 1. Introduction

Severe acute respiratory syndrome coronavirus-2 (SARS-CoV-2) is the causative agent of COVID-19, an infection that primarily targets the lungs [1]. The risk of developing severe or critical forms of COVID-19 pneumonia is influenced by underlying conditions. Notably, cardiac, vascular, and metabolic disorders including hypertension, coronary heart disease, diabetes, and obesity are highly prevalent among patients with severe COVID-19 [2,3,4,5,6], and mortality rates tend to be higher in those with cardiometabolic comorbidities [7,8]. In general, severe COVID-19 is more prevalent in patients aged greater than 50 years [2,3,8,9], a population that largely overlaps with those most at risk for or already having a history of hypertension, diabetes, or other cardiometabolic diseases [10].

In the general population, age and cardiometabolic diseases are also independently associated with the occurrence of non-COVID pneumonia [11]. Obesity has been considered an independent predisposing factor for severe H1N1 pulmonary infection [12]. Further, patients with known cardiovascular diseases are at higher risk of severe outcomes when suffering from lower respiratory tract infections, especially those related to influenza [13]. RAS blockers have been shown to improve pneumonia-related outcomes even in patients with viral [14,15] and influenza pneumonia [16].

Given the world-wide dissemination of the COVID-19 pandemic, further evidence is warranted for the appropriate management of patients with cardiometabolic disorders [17]. A limited number of studies have directly compared the factors associated with the risk of presenting severe or fatal COVID-19 with those found in patients with severe influenza pneumonia [18,19]. The aim of the present study was to compare the characteristics and prognoses of patients hospitalized for COVID-19 in 2020 with patients hospitalized for influenza during the 2018–2019 season.

## 2. Materials and Methods

### 2.1. Study Design and Participants

We conducted a single-center observational study on the first 253 consecutive adult patients (≥18 years) with laboratory-confirmed COVID-19 infection admitted to the Hôpital Européen Georges Pompidou (HEGP) in Paris, France, between 12 March and 1 April 2020 and further hospitalized for more than 24 h owing to severe or critical pneumonia, as previously defined in [7]. Confirmed COVID-19 cases were defined by positive real-time reverse-transcriptase polymerase chain reaction (RT-PCR) results for SARS-CoV-2 performed on nasopharyngeal or oropharyngeal swab specimens, bronchoalveolar lavage samples, or bronchial aspirates. COVID-19 pneumonia cases were confirmed by (i) a peripheral oxygen saturation level (SpO_2_) ≤ 94% as measured by a pulse oximeter device and/or (ii) an abnormal chest computed tomography (CT) scan with radiological patterns consistent with COVID-19 [20]. Fourteen-day mortality was monitored for all included patients.

To determine if associated characteristics were specific to patients with COVID-19, we formed a second retrospective cohort of 153 consecutive adult patients (≥18 years) admitted to the same hospital (HEGP, Paris, France) with seasonal influenza respiratory tract infections and requiring hospitalization because of hypoxia with an SpO_2_ ≤ 94%, the same criteria used for determining pneumonia in COVID-19 patients. We identified these patients with severe or critical influenza respiratory infection in the immediate period preceding the advent of the COVID-19 epidemic in the Paris area, i.e., between 8 December 2017 and 17 December 2019, using the medico-administrative data of the hospital. Patients were included if their hospital stay was classified in the influenza group of the French Diagnostic Related Group (DRG) system.

### 2.2. Data Collection

Demographic, clinical, comorbidity, treatment, and outcome data for patients with COVID-19 were extracted from the electronic medical records collected in a standardized data collection form implemented on 11 March 2020 in the Clinical Data Warehouse (CDW) of the HEGP. The dedicated medical records were stored on an i2b2 platform in the CDW along with all other hospital health records. Demographic, clinical, treatment, and outcome data for patients with influenza respiratory tract infections were also extracted from the CDW of the HEGP. All data were separately verified by two investigators separately (A.F. and M.L.) and discordances in interpretation were reconciled by a third investigator (J.S.H.).

### 2.3. Definitions

The intensive care unit (ICU) patients with COVID-19 were defined by hospitalization in one of the dedicated ICU units representing 110 ICU beds as of 1 April 2020. Indication of ICU admission was based on the presence of acute respiratory distress syndrome (ARDS) defined according to the Berlin Definition [21] and requiring high-flow nasal oxygen therapy, non-invasive mechanical ventilation, or invasive mechanical ventilation. Hospitalization in non-ICU units (representing 158 beds as of 1 April 2020) was determined when patients required only nasal, low-flow oxygen administration and standard medical monitoring. Direct admission to the ICU was defined as patients requiring immediate transfer or transfer within 24 h to an ICU (referred as critical COVID-19). Secondary admission to the ICU was defined for non-ICU patients who required ICU admission >24 h after their initial admission. SpO_2_ was measured upon admission, usually in the emergency room and before oxygen administration, when possible. Body mass index (BMI) was measured at hospital admission, and obesity was defined as a BMI ≥ 30 kg/m^2^.

Medical history and smoking status were collected at hospital admission as reported by the patient (or by the accompanying person if the patient was unable to communicate), or directly from the electronic medical record for those who had a previous admission in our institution. Information on the following variables was extracted: age, sex, any history of cardiovascular or metabolic disease (defined by pre-existing hypertension, diabetes, hypercholesterolemia, ischemic heart disease, or atrial fibrillation), pulmonary disease (chronic obstructive pulmonary disease (COPD) or asthma), chronic kidney disease, hypothyroidism, or malignancy (excluding non-melanoma skin malignancy). These comorbidities were defined according to the current French or European guidelines. Data on chronic kidney disease (estimated glomerular filtration rate (eGFR) by MDRD ≤ 45 mL min^−1^⋅73 m^−2^, grade IIIb) and hypothyroidism were retrieved from past medical histories or laboratory measurements performed prior to hospitalization. An active cancer case was defined as a patient treated for cancer, and a previous cancer case was defined as a patient cured of a malignant disease. Smoking status was defined as “never smoker”, “active smoker”, or “previous smoker (smoking cessation > 3 months)”. Cardiovascular treatments were retrieved from the patient’s prescriptions at admission, including RAS blockers, beta-blockers, diuretics, calcium channel blockers, and statins.

### 2.4. Statistical Analysis

Continuous variables were presented as means ± standard deviation (SD) while categorical variables are shown as frequency (%) unless otherwise stated. Continuous data were compared using Student’s *t*-test and categorical variables were compared using the χ^2^ or Fisher’s exact test, as appropriate.

Risk factors associated with critical COVID-19 on admission, as determined by the need for direct ICU hospitalization, were analyzed using odds ratios (ORs) and 95% confidence intervals (CIs) estimated for each variable using a logistic regression model adjusted for age and sex. Significance was assessed with the Wald test. Variables with a value of *p* < 0.25 were subjected to a multivariate logistic regression analysis to simultaneously examine their independent effect. The final age- and sex-forced model was obtained through stepwise deletion of variables until all the predictors left had a p value less than 0.05. BMI or obesity (defined as BMI ≥ 30 kg/m^2^) were tested in separate models.

Overall survival was analyzed with a two-sided log-rank test, and hazard ratios (HRs) and two-sided 95% CI values were calculated using the Cox proportional hazard model. The proportional hazard assumption was assessed by testing the interaction between covariates and time. Sex and age (dichotomized at the median value) were also forced into the Cox model.

The comparison of characteristics between patients with COVID-19 and influenza respiratory infections was performed using an OR and a 95% CI estimated for each variable using a sex- and age-adjusted logistic regression model. Any characteristic with an OR > 1 was more frequently observed in COVID-19 patients than in influenza patients. Significance was assessed by the Wald test.

All statistical analyses were performed using SAS (version 9.4; SAS Institute, Cary, NC, USA). Two-sided *p* values ≤ 0.05 were considered statistically significant.

## 3. Results

### 3.1. Patients with COVID-19

#### 3.1.1. Baseline Characteristics

**Figure 1:**
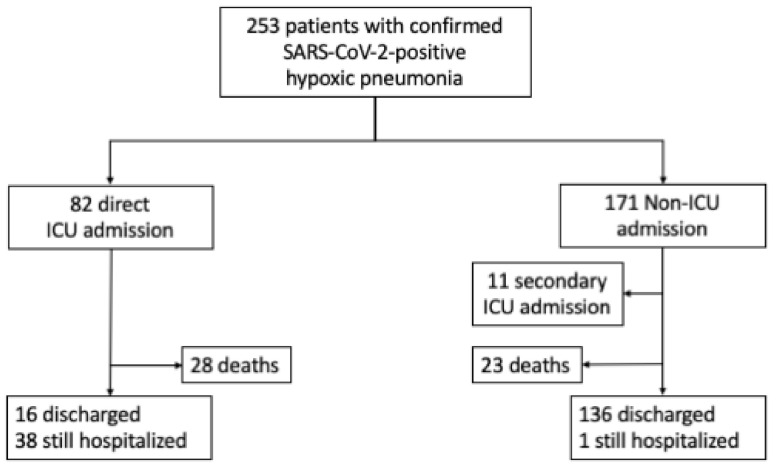
Flow diagram of patients with COVID-19 included in the study and outcomes during the 14-day follow up.

Between 12 March and 1 April 2020, 253 patients were hospitalized at the HEGP with confirmed SARS-Cov-2-positive pneumonia. Their mean age was 64.7 ± 16.1 years, and 174 patients (68.8%) were male (Table 1). A total of 82 patients were critical and required immediate or within 24 h transfer to an ICU. The remaining 171 patients with COVID-19 required non-ICU hospitalization at admission (Figure 1). The mean delay between the onset of symptoms and hospitalization was 6.9 ± 3.4 days, and there was no significant difference between the groups (7.2 ± 3.2 vs. 6.8 ± 3.5 days; *p* = 0.44 for ICU versus non-ICU, respectively).

#### 3.1.2. Risk of ICU Admission

In comparison with the non-ICU patients with COVID-19, those in the ICU were predominantly male (79.3% vs. 63.7%; *p* = 0.013) and significantly younger (61.2 ± 11.4 vs. 66.4 ± 17.7 years; *p* = 0.016) (Table 1). After adjusting for age and sex, significant differences were noted in cardiometabolic comorbidities between both groups (Table 2). In comparison with the non-ICU patients, those in the ICU had higher BMIs (adjusted odds ratio [adjOR]: 1.15; 95% CI 1.08 to 1.23; *p* < 0.0001) and, therefore, were more likely to be obese (adjOR: 2.56; 95% CI 1.37 to 4.79; *p* = 0.0031) or to have type 2 diabetes mellitus (adjOR: 2.11; 95% CI 1.10 to 4.05; *p* = 0.024). On the contrary, the ICU patients were less likely to have had a previous or active cancer diagnosis (adjOR: 0.25; 95% CI 0.08 to 0.74; *p* = 0.013). No significant differences were observed between the two groups for other cardiovascular diseases including having a history of hypertension (adjOR: 1.37; 95% CI 0.75 to 2.48; *p* = 0.30) or ischemic heart disease (adjOR: 0.86; 95% CI 0.33 to 2.25; *p* = 0.76). Moreover, no significant imbalance was noted in ongoing cardiovascular treatments at entry between these groups (Table 2). In the multivariate analysis with age and sex forced into the model, obesity (BMI ≥ 30 kg/m^2^) was the only cardiometabolic disorder independently associated with the risk of presenting critical COVID-19 that required direct ICU admission (adjOR: 2.56; 95% CI 1.37–4.79; *p* = 0.0031). This result was consistent with that of additional sensitivity analyses that excluded patients with secondary admission to the ICU (*n* = 11) or included patients with relative contraindications for ICU admission (age > 80 years or patients with cancer, *n* = 67).

#### 3.1.3. Mortality Rates and Predictors

After a 14-day follow-up, 51 deaths (20.1%) occurred during hospitalization including 28 (34.1%) deaths in ICU patients and 23 (13.4%) in non-ICU patients (adjusted HR (adjHR): 3.09; 95% CI 1.76 to 5.42; *p* < 0.0001). In the multivariate analysis with age and sex forced into the model, the risk of death was higher only among patients with a history of hypertension (adjHR: 2.22; 95% CI 1.20–4.10; *p* = 0.0111), which was the only factor independently associated with early mortality.

### 3.2. Comparison between Patients with COVID-19 and Influenza Respiratory Infection

**Figure 2:**
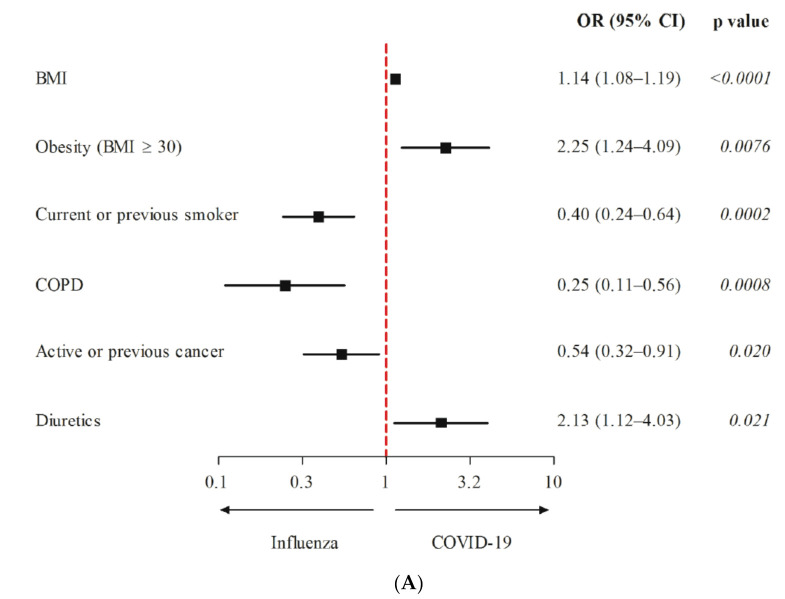

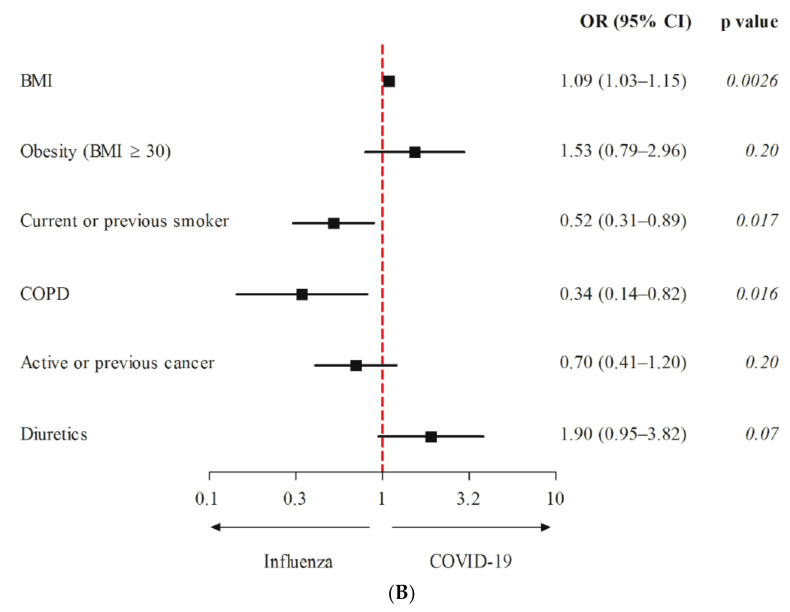
Representation of ORs between COVID-19 and influenza hospitalized patients. (**A**) ICU and non-ICU patients. (**B**) Non-ICU patients.

From 8 December 2017 to 17 December 2019, a total of 153 patients with influenza pneumonia were hospitalized at our institution (Table 1). The rate of ICU admission among these patients was much lower than that in patients with COVID-19 (5.2% vs. 32.4%, *p* < 0.0001). Patients with influenza were significantly older than those with COVID-19 and showed an equal distribution of male and female patients (Table 1). The proportion of patients with pre-existing hypertension or on antihypertensive medication was similar between the two groups, but the median (range) number of medications at entry was lower in patients with influenza than in those with COVID-19 (1 (1–4) versus 2 (1–6)). In the analysis adjusted for age and sex, patients with COVID-19 were more likely to be obese (adjOR: 2.25; 95% CI 1.24 to 4.09; *p* = 0.0076) and to receive diuretics (mainly (86.7%) hydrochlorothiazide; adjOR: 2.13; 95% CI 1.12 to 4.03; *p* = 0.021) but were less likely to be smokers (adjOR: 0.40; 95% CI 0.24 to 0.64; *p* = 0.0002), to have COPD (adjOR: 0.25; 95% CI 0.11 to 0.56; *p* = 0.0008), or have had a previous or active cancer diagnosis (adjOR: 0.54, 95% CI 0.32 to 0.91; *p* = 0.020) (Table 3 and Figure 2A). Analysis restricted to non-ICU patients gave comparable effect sizes, although some associations were no longer significant owing to the smaller sample size (Figure 2B). Similar results were found when the analysis was restricted to patients with influenza hospitalized in 2019, the closest period to the COVID-19 epidemic.

## 4. Discussion

This single-center analysis was performed on 253 initial COVID-19 patients admitted from 12 March 2020 to 1 April 2020, providing a picture of the first COVID-19 cases with severe and critical pneumonia as the SARS-CoV-2 epidemic expanded in France before the initiation of a lockdown on 16 March 2020. In this cohort, obese patients and those receiving medications for hypertension were found to be at a higher risk of critical and fatal COVID-19. These associations were significant after adjusting for age and sex. Our results highlight the typical profile of a severe or critical COVID-19 patient as a 65-year-old male with underlying metabolic syndrome. These characteristics do not seem to overlap with the classical pattern of viral respiratory infections as our data indicate that patients hospitalized at the same center for influenza pneumonia during the winters of 2017–2018 and 2018–2019 were significantly older with lower BMIs and with an equal distribution between male and female as well as having a higher proportion of underlying lung diseases as evidenced by the higher rates of COPD or reported smoking habits. This result is concordant with a higher tropism for the endothelium with SARS-CoV-2 as compared to influenza viruses [22].

In comparison with 153 patients with influenza pneumonia from 8 December 2017 to 17 December 2019 and hospitalized in the same hospital and thereby representing the same patients at risk, we confirm a recent study performed on a French national administrative database showing that the presentation of patients with COVID-19 and seasonal influenza requiring hospitalization differs significantly [18]. In our study, however, we only included an adult population whereas 8942 of the 45,819 patients (19.5%) included in the national cohort were under the age of 18, which may have influenced the associated comorbidities [18]. We also included patients hospitalized for more than 24 h to rule out asymptomatic or minor illness.

Previous case series have reported the association between obesity and COVID-19 severity [3,4,6]. Obesity was also found to be related to the severity of other respiratory infections including the H1N1 influenza [12]. However, our analysis of patients with influenza pneumonia admitted in our center indicates that this pattern is not classic to other viral respiratory diseases but rather suggests the specific predisposition of obese patients to severe COVID-19. Although this association is consistent with that reported in other communities, including China [23] and the United States [3], it has not been observed for SARS-CoV-1.

While pre-existing hypertension was previously thought to be a predisposing factor to COVID-19 [2,8,17], we failed to observe this association in our patients because the proportion of hypertensive patients was similar between COVID-19 and those with influenza cases and in line with the known prevalence of hypertension in this age category [24]. On the contrary, the association between pre-existing hypertension and the risk of short-term, in-hospital mortality was consistent with that reported in China [25]. In our study, hypertension was the only factor independently associated with in-hospital death after considering all potential confounders including age and sex. Interestingly, hypertension was more frequently reported in patients with SARS-CoV-1 requiring ICU admission during the 2003 epidemic [26]. Our results demonstrate a characteristic link between COVID-19 and hypertension that may have practical implications. Almost all COVID-19 patients with hypertension were treated with antihypertensive medications upon admission (93.2%) and received a median of two medications. In comparison, patients with influenza pneumonia received a median of only one antihypertensive medication despite being older. Thus, COVID-19 affected patients with a more advanced hypertension phenotype than those with influenza as evidenced by the pre-hospitalization prescription of more antihypertensive medications at a younger age as compared with patients with influenza. This observation indicates that a higher degree of previous and long-lasting vascular damage and endothelial dysfunction in hypertensive patients who are then affected by COVID-19 increases the potential risk of endothelial invasion by SARS-CoV-2, consistent with the association with COVID-19 severity [22]. Pre-hospitalization diuretic prescriptions were more common in patients with COVID-19 than in those with influenza pneumonia. The ongoing diuretic treatment at entry mainly comprised hydrochlorothiazide administered in combination with other antihypertensive therapies, reiterating the specific association between COVID-19 and hypertension and its treatments. These data also indicate the need for specific measures and increased surveillance of patients with cardiometabolic diseases in the context of the COVID-19 pandemic.

Histories of chronic respiratory diseases were less significantly observed in patients with COVID-19 than in those with influenza respiratory infections, suggesting the protective role of immunomodulating therapies such as inhaled corticosteroids [27]. Dedicated studies in suitable animal models and in humans are warranted to confirm these hypotheses.

Our study has some limitations. As a single-center study, the total sample size is smaller than that in other studies. However, the different patient characteristics are compatible with those observed in the French population of same age and sex [24], indicating that our data are representative of an unselected group of French patients infected with SARS-CoV-2 during the first wave of the pandemic in 2020. It is also unknown whether the influence of cardiometabolic factors is different with novel SARS-CoV-2 variants.

## 5. Conclusions

Critical COVID-19 pneumonia is associated with sex (male) and obesity while in-hospital mortality is associated with pre-existing hypertension independent of age and sex. The clinical factors favoring the incidence of COVID-19 do not overlap with those favoring influenza respiratory infections. Notably, cardiometabolic disorders differentially influenced the risk of presenting a severe COVID-19 or influenza pneumonia.

## Figures and Tables

**Table 1:** Demographic and clinical characteristics, comorbidities, and cardiovascular therapies of COVID-19 patients requiring admission to ICU and non-ICU patients hospitalized between 12 March 2020 and 1 April 2020 and of influenza respiratory infection patients requiring hospitalization (2017–2019). Reported data are at hospital admission at Hôpital Européen Georges Pompidou in Paris, France.

	COVID-19 Group			Influenza Group
	ICU (*n* = 82)	Non-ICU (*n* = 171)	Overall (*n* = 253)	Overall (*n* = 153)
**Demographic and clinical characteristics**				
Mean age (years)	61.2 (11.4)	66.4 (17.7)	64.7 (16.1)	69.8 (19.9)
Sex Male Female	65 (79.3%)17 (20.7%)	109 (63.7%) 62 (36.3%)	174 (68.8%) 79 (31.2%)	79 (51.6%) 74 (48.4%)
Body mass index (kg/m^2^)	29.7 (5.8)	26.0 (4.4)	27.4 (5.2)	24.0 (5.3)
Mean SpO_2_ (%)	87.8 (8.8)	94.4 (4.1)	92.3 (6.8)	93.8 (5.2)
**Cardiometabolic diseases and risk factors**				
Hypertension On-treatment Number of medications *	39 (47.6%)	80 (46.8%)	119 (47.0%)	70 (45.8%)
	35/39 (89.7%)	75/80 (94.9%)	110/119 (93.2%)	67/70 (95.7%)
	2 (1–5)	2 (1–6)	2 (1–6)	1 (1–4)
Type 2 diabetes	23 (28.0%)	28 (16.4%)	51 (20.2%)	28 (18.3%)
Obesity (BMI ≥ 30 kg/m^2^)	34/82 (41.5%)	30/139 (21.2%)	64/221 (29.0%)	18/121 (14.9%)
Current or previous smoker	15/77 (19.5%)	42/151 (26.1%)	57/228 (23.9%)	52/123 (42.3%)
Hypercholesterolemia	18 (22.2%)	47 (27.5%)	65 (25.8%)	52 (34.0%)
Ischaemic heart disease	7 (8.5%)	19 (11.1%)	26 (10.3%)	24 (15.7%)
Atrial fibrillation	4 (4.9%)	21 (12.3%)	25 (9.9%)	26 (17.0%)
Chronic kidney disease	4 (4.9%)	19 (11.1%)	23 (9.1%)	22 (14.4%)
**History of other comorbidities**				
Asthma	6 (7.3%)	10 (5.8%)	16 (6.3%)	16 (10.5%)
COPD	1 (1.2%)	8 (4.7%)	9 (3.6%)	22 (14.4%)
Hypothyroidism	5 (6.1%)	9 (5.3%)	14 (5.5%)	17 (11.1%)
Active or previous cancer	4 (4.9%)	34 (19.9%)	38 (15.0%)	43 (28.1%)
**Cardiovascular therapies at entry among all patients**				
ACEi	10 (12.2%)	14 (8.2%)	24 (9.5%)	20 (13.1%)
ARB	21 (25.6%)	35 (20.5%)	56 (22.2%)	26 (17.0%)
ACEi or ARB	31 (37.8%)	49 (28.8%)	80 (31.7%)	45 (29.4%)
Diuretics	17 (20.7%)	29 (16.5%)	45 (17.9%)	15 (9.8%)
Beta-blockers	7 (8.5%)	26 (15.3%)	33 (13.1%)	13 (8.5%)
Calcium channel blockers	16 (19.5%)	33 (19.4%)	49 (19.4%)	27 (17.6%)
Statins	16 (19.5%)	42 (24.7%)	58 (23.0%)	47 (30.9%)

Data are expressed as mean (SD) or *n* (%). *: median (range) ICU: intensive care unit; BMI: body mass index; COPD: chronic obstructive pulmonary disease; ACEi: angiotensin-converting enzyme inhibitors; ARB: angiotensin II receptor blocker; SpO_2_: peripheral oxygen saturation.

**Table 2:** Risk factors of COVID-19 severity among hospitalized patients with COVID-19 as determined from the need for ICU admission.

	Adjusted * OR (95% CI)	*p* Value
**Demographic and clinical presentation**		
BMI (per 1 kg/m^2^ increase)	1.15 (1.08–1.23)	<0.0001
Mean SpO_2_ (per 1% increase)	0.83 (0.77–0.88)	<0.0001
**Cardiometabolic diseases and risk factors**		
Hypertension	1.37 (0.75–2.48)	0.30
Type 2 diabetes	2.11 (1.10–4.05)	0.024
Obesity (BMI ≥ 30 kg/m^2^)	2.56 (1.37–4.79)	0.0031
Current or previous smoker	0.74 (0.37–1.48)	0.39
Hypercholesterolemia	0.85 (0.44–1.63)	0.62
Ischemic heart disease	0.86 (0.33–2.25)	0.76
Atrial fibrillation	0.41 (0.13–1.26)	0.12
Chronic kidney disease	0.43 (0.14–1.33)	0.14
**History of other comorbidities**		
Asthma	1.24 (0.42–3.66)	0.70
COPD	0.31 (0.04–2.57)	0.28
Hypothyroidism	1.58 (0.49–5.15)	0.44
Active or previous cancer	0.25 (0.08–0.74)	0.013
**Cardiovascular therapies at entry**		
ACEi	1.64 (0.68–3.98)	0.27
ARB	1.57 (0.81–3.06)	0.18
ACEi or ARB	1.83 (0.99–3.37)	0.053
Diuretics	1.42 (0.71–2.84)	0.32
Beta-blockers	0.53 (0.21–1.29)	0.16
Calcium channel blockers	1.08 (0.54–2.15)	0.82
Statins	0.84 (0.43–1.64)	0.60

ICU: intensive care unit; OR: odds ratio; BMI: body mass index; COPD: chronic obstructive pulmonary disease; ACEi: angiotensin-converting enzyme inhibitors; ARB: angiotensin II receptor blocker; SpO_2_: peripheral oxygen saturation; CI: confidence interval * OR values are adjusted for sex and age. OR > 1 indicates that the characteristic was more frequently observed in COVID-19 patients requiring ICU hospitalization than in other hospitalized COVID-19 patients. Bold: subtitle.

**Table 3:** Comparison of characteristics between patients hospitalized with COVID-19 and influenza.

	Adjusted * OR (95% CI)	*p* Value
**Demographic and clinical presentation**		
BMI (per 1 kg/m^2^ increase)	1.14 (1.08–1.19)	<0.0001
Mean SpO_2_ (per 1% increase)	0.96 (0.92–1.00)	0.037
**Cardiometabolic diseases and risk factors**		
Hypertension	1.24 (0.80–1.93)	0.33
Type 2 diabetes	1.12 (0.66–1.90)	0.67
Obesity (BMI ≥ 30 kg/m^2^)	2.25 (1.24–4.09)	0.0076
Current or previous smoker	0.40 (0.24–0.64)	0.0002
Hypercholesterolemia	0.69 (0.44–1.10)	0.12
Ischemic heart disease	0.59 (0.31–1.12)	0.11
Atrial fibrillation	0.58 (0.31–1.08)	0.08
Chronic kidney disease	0.54 (0.28–1.02)	0.06
**History of other comorbidities**		
Asthma	0.53 (0.25–1.14)	0.10
COPD	0.25 (0.11–0.56)	0.0008
Hypothyroidism	0.59 (0.28–1.26)	0.17
Active or previous cancer	0.54 (0.32–0.91)	0.020
**Cardiovascular therapies at entry**		
ACEi	0.70 (0.37–1.33)	0.28
ARB	1.57 (0.92–2.69)	0.10
ACEi or ARB	1.21 (0.76–1.91)	0.42
Diuretics	2.13 (1.12–4.03)	0.021
Beta-blockers	1.70 (0.85–3.41)	0.13
Calcium channel blockers	1.19 (0.70–2.04)	0.52
Statins	0.70 (0.44–1.12)	0.13

OR: odds ratio; BMI: body mass index; COPD: chronic obstructive pulmonary disease; ACEi: angiotensin-converting enzyme inhibitors; ARB: angiotensin II receptor blocker; SpO_2_: peripheral oxygen saturation; CI: confidence interval. * OR values are adjusted for sex and age. OR > 1 indicates that the characteristic is more frequent in COVID-19 group than in influenza group. Bold: subtitle.

## Data Availability

The data presented in this study are available on request from the corresponding author. The data are not publicly available due to privacy restrictions.
